# Psychometric properties of the chinese version of multidimensional experiential avoidance questionnaire-30

**DOI:** 10.1186/s40359-024-01790-x

**Published:** 2024-05-24

**Authors:** Dongdong Xue, Hongxing Meng, Hongpei Liu, Nana Wang, Jin He, Lina Feng, Juan Su, Xiaozhuang Wang

**Affiliations:** 1 Faculty of Psychology, Tianjin Normal University, Tianjin, 300387 China; 2 Xinquan Primary School, Zengcheng District, Guangzhou, China; 3 Tianjin Medical University Cancer Institute and Hospital, Tianjin, China; 4 Key Research Base of Humanities and Social Sciences of the Ministry of Education, Academy of Psychology and Behavior, Tianjin Normal University, Tianjin, 300387 China; 5 Tianjin Social Science Laboratory of Students’ Mental Development and Learning, Tianjin, 300387 China

**Keywords:** Experiential avoidance, Classical test theory, Multidimensional item response theory, Measurement invariance

## Abstract

**Background:**

Experiential avoidance represents the tendency to avoid negative internal experiences, which is a key concept in Acceptance and Commitment Therapy. However, existing measures of experiential avoidance (i.e., Acceptance and Action Questionnaire–II, AAQ-II) have some limitations. This study aims to assess the psychometric properties of the Chinese version of Multidimensional Experiential Avoidance Questionnaire-30 (MEAQ-30) and provide evidence for the reliability and validity of this new instrument.

**Methods:**

Two questionnaire surveys were conducted. The first sample (*N* = 546) was analyzed using classical test theory (CTT), and the second sample (*N* = 511) was analyzed using multidimensional item response theory (MIRT).

**Results:**

CTT supported the six-factor structure of MEAQ-30, indicating good internal consistency and measurement invariance across genders. Furthermore, the Chinese version of MEAQ-30 showed satisfactory convergent and discriminant validity. The incremental validity test showed that after controlling for the effects of neuroticism and AAQ-II, the Chinese version of MEAQ-30 could still significantly predict depression, anxiety, and stress. MIRT indicated that 30 items had good discrimination and difficulty, and the six subscales were sufficiently reliable across the continuum of experiential avoidance.

**Conclusion:**

The Chinese version of MEAQ-30 has good reliability and validity and is suitable for assessing experiential avoidance among Chinese populations.

**Supplementary Information:**

The online version contains supplementary material available at 10.1186/s40359-024-01790-x.

## Introduction

Experiential avoidance (EA) is defined as the intentional efforts to evade distressing emotions, thoughts, and other private experiences [1, 2]. Although avoiding negative experiences is a natural psychological phenomenon in humans, excessive avoidance of distressing experiences can be counterproductive, which in turn can lead to a range of mental health problems [1, 3]. Numerous studies have demonstrated that high levels of EA are associated with various diagnostic categories, such as anxiety disorder [4], depression disorder [5], and posttraumatic stress disorder [6]. In recent years, there has been a growing interest in studying EA among Chinese populations [7–9]. However, the existing Chinese EA measures are relatively single and vaguely conceptualized, failing to cover the multiple constructs of EA adequately and limiting the validity of EA measures. Therefore, this study aimed to assess the psychometric properties of a new instrument for measuring EA and to provide a more valid measurement for EA-related research in China.

Multiple self-report measures have been created to assess EA. The Acceptance and Action Questionnaire (AAQ-I) is a single-factor instrument initially designed to measure EA, yet it exhibited relatively low internal consistency and retest reliability [10]. Subsequently, Bond et al. [11] revised the AAQ-I to develop the Acceptance and Action Questionnaire (AAQ-II) to address these shortcomings. Like the AAQ-I, the AAQ-II is a single-factor scale. It consists of seven items, each scored on a seven-point scale, with commendable reliability and validity [11]. So far, AAQ-II has found widespread use in Acceptance and Commitment Therapy (ACT) research and has been translated into multiple languages for diverse populations [3, 12, 13]. However, AAQ-II also has some limitations that cannot be ignored. Several studies have indicated that the AAQ-II exhibits low discriminant validity concerning neuroticism, negative affect, and psychological distress [14–16]. Moreover, the unidimensional nature of the AAQ-II fails to capture the full spectrum of EA [3], which may obscure variations in different avoidance tendencies, thereby potentially overlooking valuable and predictive information [17].

Given the broad scope of behaviors, thoughts, and emotions encompassed by EA [1], Gámez et al. [18] developed the Multidimensional Experiential Avoidance Questionnaire (MEAQ) to facilitate the multidimensional assessment of EA. Specifically, the MEAQ comprises 62 items distributed across six dimensions: behavioral avoidance, distress aversion, procrastination, distraction/suppression, repression/denial, and distress endurance. Each dimension represents a distinct behavioral aspect of avoiding distressing experiences. Previous research has consistently demonstrated that MEAQ showed better discriminant validity and reliability than the AAQ-II [18–20]. The multidimensional assessment of EA can help identify potential associations between different facets of EA and various types of psychopathology [21]. However, the MEAQ’s extensive item count limits its practical application. Psychotherapy research requires concise measures to facilitate the repeated assessment of constructs [22].

To simplify the multidimensional assessment of EA, Sahdra et al. [20] streamlined the MEAQ items using genetic algorithms, resulting in the development of the Multidimensional Experiential Avoidance Questionnaire-30 (MEAQ-30). Genetic algorithms are machine learning approaches that mimic Darwinian evolution principles to abbreviate scale in a fully automated manner and maximize the captures of the variance in the original data [20, 23]. MEAQ-30 retained the six dimensions of the original questionnaire and has nearly identical psychometric properties to MEAQ [20], significantly reducing the cognitive burden on participants. To date, MEAQ-30 has been widely used in EA-related fields [19, 24, 25] and translated from English to Turkish [26], which has proved to have sufficient reliability and validity [19, 26]. However, MEAQ-30 has yet to undergo validation in the Chinese context.

Most of the existing studies still use the AAQ-II to assess EA among Chinese populations [7–9] and do not adequately consider the limitations of this instrument, which limits the validity of EA measures. Compared to AAQ-II and MEAQ, MEAQ-30 is undoubtedly a more promising tool, which helps to assess the multidimensional structure of EA quickly. Moreover, few studies have examined the applicability of the multidimensional structure of EA in Eastern cultures. A growing body of studies has shown that EA is influenced by culture and values [3, 27, 28]. Given that Chinese and Western cultures have different perspectives on distress, this may contribute to differences in the expression of EA [3]. Therefore, validating the psychometric properties of the Chinese version of MEAQ-30 will contribute to a clearer understanding of the potential impact of cultural differences on EA and provide a new instrument for advancing research on EA in China.

The current study aims to employ Classical Test Theory (CTT) and Multidimensional Item Response Theory (MIRT) to analyze the psychometric properties of the Chinese version of MEAQ-30. Previous studies have utilized CTT to evaluate the reliability and validity of EA instruments [21, 27, 28]. However, CTT relies on sample-specific estimates for item parameters and reliability, which may not accurately reflect the true level of the trait. On the other hand, Item Response Theory (IRT) models can assess the relationship between item response and latent trait, offering insights into the different levels of the latent trait for each item [29]. Traditional IRT models are designed for single latent trait instruments, posing challenges in applying unidimensional IRT models to multidimensional data. Multidimensional Item Response Theory (MIRT) models were introduced to overcome this limitation, allowing the simultaneous measurement of multiple traits [30]. The multidimensional structure of MEAQ-30 lends itself well to MIRT analysis, enabling the concurrent consideration of correlations between different subscales. To the best of our knowledge, no studies have been conducted to analyze MEAQ-30’s item performance through MIRT.

Taken together, this study aimed to comprehensively assess the psychometric properties of the Chinese version of the MEAQ-30 using both CTT and MIRT. First, the factor structure and internal consistency of the Chinese version of MEAQ-30 were examined. In addition, previous studies did not perform measurement invariance analysis of the MEAQ-30. The current study tested measurement invariance of the MEAQ-30 across genders, examining whether it functions equivalently in males and females. Second, testing the convergent validity and discriminant validity of the MEAQ-30. Considering that existing EA measures (e.g., AAQ-II) overlap with other related concepts (e.g., neuroticism), resulting in poor discriminant validity. Therefore, the current study examined the incremental predictive power of the MEAQ-30 for psychological distress (e.g., depression, anxiety, and stress) after controlling for the effects of neuroticism and AAQ-II. Third, the MIRT was used to analyze item performance (e.g., discrimination and difficulty) in the MEAQ-30 to determine how each item provides information at different levels of EA.

## Methods

### Participants

The current study conducted two online surveys via the Internet. All participants completed an informed consent form, and the responses were anonymous.

The first survey was conducted between December 2022 and March 2023 and included the Chinese version of MEAQ-30 and other criteria variables (e.g., AAQ-II, Neuroticism, DASS-21). There were 546 participants in this sample from China, ages 18 to 32 (*M* = 21.36, *SD* = 1.99). Among the participants, 44.14% were male, 55.86% were female, 84.98% were college students, and 15.02% were professional employees.

The second survey was conducted between April 2023 and June 2023 and included the Chinese version of MEAQ-30. There were 511 participants in this sample from China, ages 17 to 38 (*M* = 22.45, *SD* = 3.59). Among the participants, 43.60% were male, 56.40% were female, 86.70% were college students, and 13.30% were professional employees.

### Procedures

The primary author of this study contacted the original author of the MEAQ-30 [20] and secured permission to adapt the questionnaire. The forward-backwards translation method was employed to translate the MEAQ-30 into Chinese. Two independent translators translated the MEAQ-30 into Chinese, and after discussions and consensus, an initial draft of the Chinese version of the MEAQ-30 was established. Subsequently, two native English speakers were engaged to translate the Chinese version of the MEAQ-30 back into English. The differences between the original and back-translated versions were discussed in depth until all translators and researchers reached a consensus. Additional file 1 provided the item content for MEAQ-30 (Appendix A is the English version of MEAQ-30, and Appendix B is the Chinese version of MEAQ-30).

### Measure

#### Multidimensional experiential avoidance Questionnaire-30 (MEAQ-30) [20]

The MEAQ-30 comprises six subscales: behavioral avoidance, distress aversion, procrastination, distraction/suppression, repression/denial, and distress endurance. Response options for its 30 items range from 1 (strongly disagree) to 6 (strongly agree). Higher scores indicate higher levels of avoidance tendency in the respective subscale. In the current study, Cronbach’s α of the MEAQ-30 was 0.89.

#### Acceptance and action questionnaire-II (AAQ-II) [11]

The AAQ-II is a 7-item scale to measure experiential avoidance/psychological inflexibility (e.g., “I worry about not being able to control my worries and feelings”). Participants responded on a scale of 1 (never true) to 7 (always true). The higher the questionnaire score, the higher the level of experiential avoidance/psychological inflexibility. The Chinese version of the AAQ-II has previously demonstrated satisfactory internal consistency [31]. In the present study, Cronbach’s α of the AAQ-II was 0.93.

#### Chinese big five personality inventory brief version (CBFPIB) [32]: neuroticism scale

The Neuroticism scale within the CBFPIB comprises eight items (e.g., “I often worry about things that don’t matter”). Participants responded on a scale ranging from 1 (disagree strongly) to 6 (agree strongly). The higher the questionnaire score, the higher the level of neuroticism. In the current study, Cronbach’s α of the Neuroticism scale was 0.91.

#### Depression anxiety stress Scale-21 (DASS‑21) [33]

The DASS-21 was utilized to assess psychological distress (e.g., “I felt close to panic”). Responses were gathered on a scale ranging from 0 (did not apply to me at all) to 3 (applied to me very much or most of the time), where higher scores indicate elevated levels of psychological distress. The Chinese version of the DASS-21 has demonstrated satisfactory internal consistency [34]. In the present study, the Cronbach’s α of the DASS-21 was 0.96.

### Data analysis

CTT analysis was performed on the first sample (*N* = 546) using JASP software (version 0.16.4). First, the mean, standard deviation, skewness, kurtosis, and item-total correlation of the MEAQ-30 items were calculated. Second, the factor structure of the MEAQ-30 was evaluated using confirmatory factor analysis (CFA) and maximum likelihood. Model fit was assessed using the χ²/df ratio, the Comparative Fit Index (CFI), the Tucker-Lewis Index (TLI), the Root-Mean-Squared Error of Approximation (RMSEA), and the Standardized Root Mean Square Residual (SRMR). Acceptable fit criteria include a χ²/ df ratio ≤ 3, CFI and TLI close to or greater than 0.90, and RMSEA and SRMR lower than or equal to 0.08 [35]. Third, the internal consistency of the MEAQ-30 was assessed using Cronbach’s α coefficient. Values exceeding 0.70 for α indicate good reliability. Fourth, measurement invariance across gender was tested through multigroup CFA analysis, examining configural, metric, and scalar invariance models. Established criteria for invariance included ΔCFI < 0.004 and ΔRMSEA < 0.050 [36]. Fifth, the convergent validity and discriminant validity of MEAQ-30 are tested using the Pearson correlation, and the incremental validity of MEAQ-30 is tested using hierarchal regression.

MIRT analysis was performed on the second sample (*N* = 511) in this study. The MIRT was implemented using the R package ‘‘mirt’’ [37]. Given the multidimensional structure of the MEAQ-30, the Multidimensional Graded Response Model (M-GRM) [38] was employed. The analysis proceeded as follows: First, the M-GRM’s goodness of fit was assessed using the M2^∗^ statistic [39] and its associated RMSEA value. Additional fit indices, such as CFI and TLI, were also obtained. Second, the M-GRM was employed to estimate the discrimination parameter and difficulty parameters of each item. Items with discrimination parameters below 0.24 and above 3.00 may be problematic [40]. Each item included five difficulty parameters (b1, b2, b3, b4, b5) in line with the MEAQ-30 6-point Likert scale. Positive (b) values implied more difficulty, negative (b) values indicated less difficulty, and values close to zero indicated moderate difficulty [41]. Third, the item characteristics curves (ICC), item information curves (IIC), and test information curves (TIC) were presented for a comprehensive understanding.

## Results

### Descriptive statistics and item-total correlations

Table 1 presents the descriptive statistics and item-total correlations for the MEAQ-30 items. The skewness values for the 30 items ranged from − 1.19 to 0.90, and kurtosis ranged from − 1.16 to 2.24. With an absolute value of skewness less than 3 and an absolute value of kurtosis less than 10, it can be concluded that the data is generally acceptable as a normal distribution [42]. The item-total correlations ranged from 0.33 to 0.77, greater than the recommended cut point of 0.30 [43].

**Table 1:** Descriptive statistics and item-total correlations (*N* = 546)

Item	M	SD	Skewness	Kurtosis	item-total correlations
Item1	4.47	1.17	-1.07	0.97	0.62
Item2	3.58	1.45	-0.12	-1.00	0.59
Item3	4.17	1.33	-0.55	-0.39	0.71
Item4	4.28	1.31	-0.82	0.12	0.76
Item5	4.32	1.33	-0.92	0.18	0.72
Item6	4.29	1.48	-0.65	-0.53	0.46
Item7	3.19	1.52	0.29	-1.03	0.52
Item8	3.76	1.43	-0.32	-0.80	0.65
Item9	3.91	1.37	-0.23	-0.79	0.56
Item10	3.52	1.39	-0.02	-0.78	0.62
Item11	3.85	1.52	-0.40	-0.93	0.72
Item12	3.69	1.54	-0.32	-1.05	0.69
Item13	3.76	1.46	-0.39	-0.89	0.75
Item14	3.47	1.54	-0.06	-1.14	0.73
Item15	2.53	1.19	0.85	0.25	0.33
Item16	4.57	0.98	-1.19	2.24	0.59
Item17	4.67	1.04	-0.96	1.29	0.63
Item18	4.66	1.00	-1.01	1.77	0.42
Item19	4.63	1.01	-1.01	1.44	0.56
Item20	4.54	1.07	-0.80	0.79	0.53
Item21	3.42	1.53	-0.01	-1.16	0.59
Item22	3.01	1.46	0.38	-0.96	0.73
Item23	3.25	1.50	0.14	-1.08	0.72
Item24	2.97	1.48	0.46	-0.84	0.71
Item25	3.13	1.50	0.23	-1.07	0.77
Item26	2.65	1.12	0.84	0.58	0.47
Item27	2.45	1.01	0.75	0.96	0.58
Item28	2.46	0.99	0.81	1.11	0.65
Item29	2.38	1.04	0.74	0.64	0.67
Item30	2.23	0.95	0.90	1.32	0.61

### Confirmatory factor analysis

The fit values for the six-factor measurement model with the MEAQ-30 indicated good fit (χ ² (390) = 893.644, χ²/ df = 2.291, CFI = 0.929, TLI = 0.921, RMSEA = 0.049, 90% CI [0.044, 0.053], SRMR = 0.057). These findings demonstrated that the MEAQ-30 exhibits good structural validity. Except for item 15, the standardized factor loadings of the other 29 items are all greater than 0.40 (0.50 to 0.84). Item 15 (“I try to deal with problems right away”) belongs to the procrastination subscale and had a relatively low standardized loading (0.34). It should be noted that the content of item 15 is related to procrastination, but its reverse wording could easily lead to acquiescence bias among participants [44]. Moreover, previous studies have shown that adding appropriate reversed items would facilitate a comprehensive assessment of the underlying construct to be measured [45]. Considering that the standardized loading of item 15 was still greater than 0.3, it was decided not to delete this item to ensure consistency with the original questionnaire. The six-factor structure diagram of the Chinese version of MEAQ-30 can be found in Additional File 2.

### Measurement invariance across genders

The results for measurement invariance are presented in Table 2. The six-factor structure of the MEAQ-30 fited well in males (*N* = 241) and females (*N* = 305). For gender, the configural model yielded an acceptable fit (CFI = 0.915; RMSEA = 0.054). The metric invariance model demonstrated adequate fit (ΔCFI = 0.000; ΔRMSEA = 0.001). The scalar invariance model also supported measurement invariance (ΔCFI = 0.002; ΔRMSEA = 0.000). Thus, these results suggested that the items of the Chinese version of MEAQ-30 operate nearly identically across genders.

**Table 2:** Measurement invariance across genders (*N* = 546)

Model	χ^2^ (df)	CFI	RMSEA	∆CFI	∆RMSEA
Male (*N* = 241)	631.727 (390)	0.921	0.051		
Female (*N* = 305)	760.088 (390)	0.911	0.056		
Configural	1391.815 (774)	0.915	0.054		
Metric	1420.201 (804)	0.915	0.053	0.000	0.001
Scalar	1453.322 (828)	0.913	0.053	0.002	0.000

### Internal consistency

Cronbach’s α values for the total MEAQ-30 and its subscales ranged from 0.77 to 0.89 (see Table 3). These results suggested that the Chinese version of MEAQ-30 has satisfactory internal consistency.

**Table 3:** Reliability of the Chinese version of MEAQ-30 (*N* = 546)

Scale	Items	Cronbach’s α
Behavioral avoidance	1, 2, 3, 4, 5	0.86
Distress aversion	6, 7, 8, 9, 10	0.78
Procrastination	11, 12, 13, 14, 15	0.84
Distraction/suppression	16, 17, 18, 19, 20	0.77
Repression/denial	21, 22, 23, 24, 25	0.88
Distress endurance	26, 27, 28, 29, 30	0.81
MEAQ-30	——	0.89

### Convergent and discriminant validity

Pearson correlation analysis was employed to assess the convergent and discriminant validity of the MEAQ-30 (see Table 4). The Chinese version of MEAQ-30 and its six dimensions were significantly positively correlated with the AAQ-II. Except for distraction/suppression, MEAQ-30 and its five dimensions were significantly positively correlated with neuroticism, depression, anxiety, and stress. These results indicated that the Chinese version of MEAQ-30 has good convergent and discriminant validity.

**Table 4:** Correlations of the Chinese version of MEAQ-30 with other scales (*N* = 546)

Variables	1	2	3	4	5	6	7	8	9	10	11	12
1.MEAQ-30	—											
2.BehAvd	0.74^***^	—										
3.DisAvr	0.74^***^	0.51^***^	—									
4.Procst	0.76^***^	0.44^***^	0.39^***^	—								
5.DstSup	0.33^***^	0.21^***^	0.32^***^	0.04	—							
6.RepDny	0.75^***^	0.36^***^	0.41^***^	0.52^***^	0.14^***^	—						
7.DisEndr	0.30^***^	0.15^***^	0.03	0.25^***^	− 0.37^***^	0.14^**^	—					
8.AAQ-II	0.66^***^	0.41^***^	0.42^***^	0.59^***^	0.09^*^	0.60^***^	0.23^***^	—				
9.CBF-N	0.60^***^	0.38^***^	0.28^***^	0.61^***^	0.01	0.56^***^	0.26^***^	0.82^***^	—			
10.DASS-D	0.51^***^	0.24^***^	0.32^***^	0.45^***^	0.03	0.54^***^	0.21^***^	0.66^***^	0.67^***^	—		
11.DASS-A	0.50^***^	0.23^***^	0.32^***^	0.41^***^	0.07	0.57^***^	0.14^***^	0.66^***^	0.69^***^	0.85^**^	—	
12.DASS-S	0.56^***^	0.31^***^	0.35^***^	0.47^***^	0.11^***^	0.58^***^	0.14^**^	0.71^***^	0.75^***^	0.84^**^	0.86^**^	—

*Note* BehAvd = behavioral avoidance; DisAvr = distress aversion; Procst = procrastination; DstSup = distraction/suppression; RepDny = repression/denial; DisEndr = distress endurance; CBF-N = neuroticism scale; DASS-D = depression, DASS-A = anxiety, DASS-S = stress; * *p* < 0.05, ** *p* < 0.01, *** *p* < 0.001

### Incremental validity

The incremental validity of the MEAQ-30 was tested by hierarchical regression. As shown in Table 5, after controlling for the effects of neuroticism and AAQ-II, MEAQ-30 still had significant effects on depression, anxiety, and stress, with ∆R^2^ ranging from 5 to 8%. These results suggested that MEAQ-30 is an independent predictor for psychological distress (depression, anxiety, and stress) compared to neuroticism and AAQ-II.

**Table 5:** Incremental validity of the Chinese version of MEAQ-30 (*N* = 546)

Variables	B	SE	b	T	*R* ^2^	∆*R*^2^
DASS-depression						
Step-1					0.44^***^	
Neuroticism	0.26^***^	0.02	0.56^***^	13.13		
AAQ-II	0.03^**^	0.01	0.14^**^	3.25		
Step-2					0.49^***^	0.05^***^
Neuroticism	0.13^***^	0.03	0.28^***^	4.88		
AAQ-II	0.02^*^	0.01	0.10^*^	2.39		
MEAQ-30	0.18^***^	0.03	0.38^***^	7.06		
DASS-anxiety						
Step-1					0.45^***^	
Neuroticism	0.27^***^	0.02	0.59^***^	13.89		
AAQ-II	0.03^**^	0.01	0.11^**^	2.60		
Step-2					0.50^***^	0.06^***^
Neuroticism	0.13^***^	0.03	0.28^***^	4.97		
AAQ-II	0.02	0.01	0.07	1.63		
MEAQ-30	0.20^***^	0.03	0.42^***^	7.86		
DASS-stress						
Step-1					0.52^***^	
Neuroticism	0.30^***^	0.02	0.61^***^	15.23		
AAQ-II	0.04^***^	0.01	0.16^***^	4.02		
Step-2					0.60^***^	0.08^***^
Neuroticism	0.11^***^	0.03	0.23^***^	4.60		
AAQ-II	0.03^**^	0.01	0.11^**^	2.91		
MEAQ-30	0.26^***^	0.02	0.50^***^	10.56		

*Note* * *p* < 0.05,** *p* < 0.01, *** *p* < 0.001

### M-GRM model fit

MIRT analysis was performed on the second sample (*N* = 511) in this study. At the overall model level, the fitting results of the M-GRM were deemed satisfactory (CFI = 0.925, TLI = 0.913, RMSEA = 0.055, 95% CI [0.050, 0.060]).

### Item parameter

Table 6 provided IRT parameter estimates for the six subscales. For behavioral avoidance, the discrimination parameter ranged from 1.63 to 2.30. Distress aversion, the values ranged from 0.95 to 1.81. Procrastination exhibited values ranging from 0.80 to 2.95. Distraction/suppression had values ranging from 1.28 to 1.98. Repression/denial demonstrated values ranging from 1.78 to 2.31. Distress endurance showed values ranging from 1.20 to 2.49. In summary, these findings indicated that all items of the MEAQ-30 exhibited an excellent capacity to discriminate the experiential avoidance dimension. Additionally, all MEAQ-30 items reported difficulty parameters that progressed from less to more difficulty. The difficulty parameters of items were widely distributed, with most items ranging from − 3 to 3. Item 15 had a low discrimination parameter, with some difficulty parameters greater than 3, indicating that this item may be problematic.

**Table 6:** Multidimensional Graded Response Model item parameter estimates (*N* = 511)

Item	a1	a2	a3	a4	a5	a6	b1	b2	b3	b4	b5
Item1	1.68						-3.37	-2.09	-1.43	-0.30	1.36
Item2	1.63						-2.25	-1.22	-0.26	0.81	1.95
Item3	2.30						-2.14	-1.51	-0.64	0.19	1.39
Item4	1.97						-2.56	-1.74	-0.86	0.14	1.38
Item5	1.67						-2.78	-1.87	-1.20	0.05	1.55
Item6		0.95					-4.23	-2.26	-1.17	-0.12	1.66
Item7		1.22					-1.85	-0.55	0.31	1.06	2.15
Item8		1.80					-2.00	-1.15	-0.40	0.58	1.75
Item9		1.43					-3.05	-1.56	-0.55	0.44	1.88
Item10		1.81					-2.13	-1.00	-0.13	0.71	1.97
Item11			2.17				-2.09	-0.95	-0.39	0.49	1.60
Item12			2.25				-1.56	-0.74	-0.16	0.61	1.55
Item13			2.32				-1.93	-1.04	-0.39	0.45	1.65
Item14			2.95				-1.32	-0.46	0.11	0.59	1.44
Item15			0.80				-2.39	0.59	1.96	4.05	6.75
Item16				1.98			-3.15	-2.44	-1.84	-0.41	1.39
Item17				1.59			-4.01	-3.02	-1.96	-0.47	1.14
Item18				1.28			-4.07	-3.21	-2.47	-0.65	1.61
Item19				1.88			-3.36	-2.24	-1.52	-0.23	1.36
Item20				1.39			-3.29	-2.50	-1.55	-0.21	1.71
Item21					1.78		-1.95	-0.64	0.11	0.84	2.15
Item22					2.31		-1.22	-0.16	0.46	1.10	2.16
Item23					2.14		-1.65	-0.67	0.20	0.99	2.17
Item24					2.09		-1.51	-0.16	0.59	1.13	1.97
Item25					2.06		-1.48	-0.49	0.24	1.14	2.11
Item26						1.20	-1.88	0.37	1.71	2.61	3.71
Item27						1.52	-1.64	0.13	1.63	2.65	3.79
Item28						1.96	-1.27	0.28	1.61	2.46	3.78
Item29						2.49	-0.96	0.24	1.35	2.18	3.40
Item30						2.02	-0.86	0.49	1.61	2.50	3.49

*Note* a1 to a6 represent the discrimination parameters of the dimension to which the item belongs; a1 = behavioral avoidance; a2 = distress aversion; a3 = procrastination; a4 = distraction/suppression; a5 = repression/denial; a6 = distress endurance; b1 to b6 represent the difficulty parameter of the items

### Item characteristics curves (ICC)

**Fig. 1:**
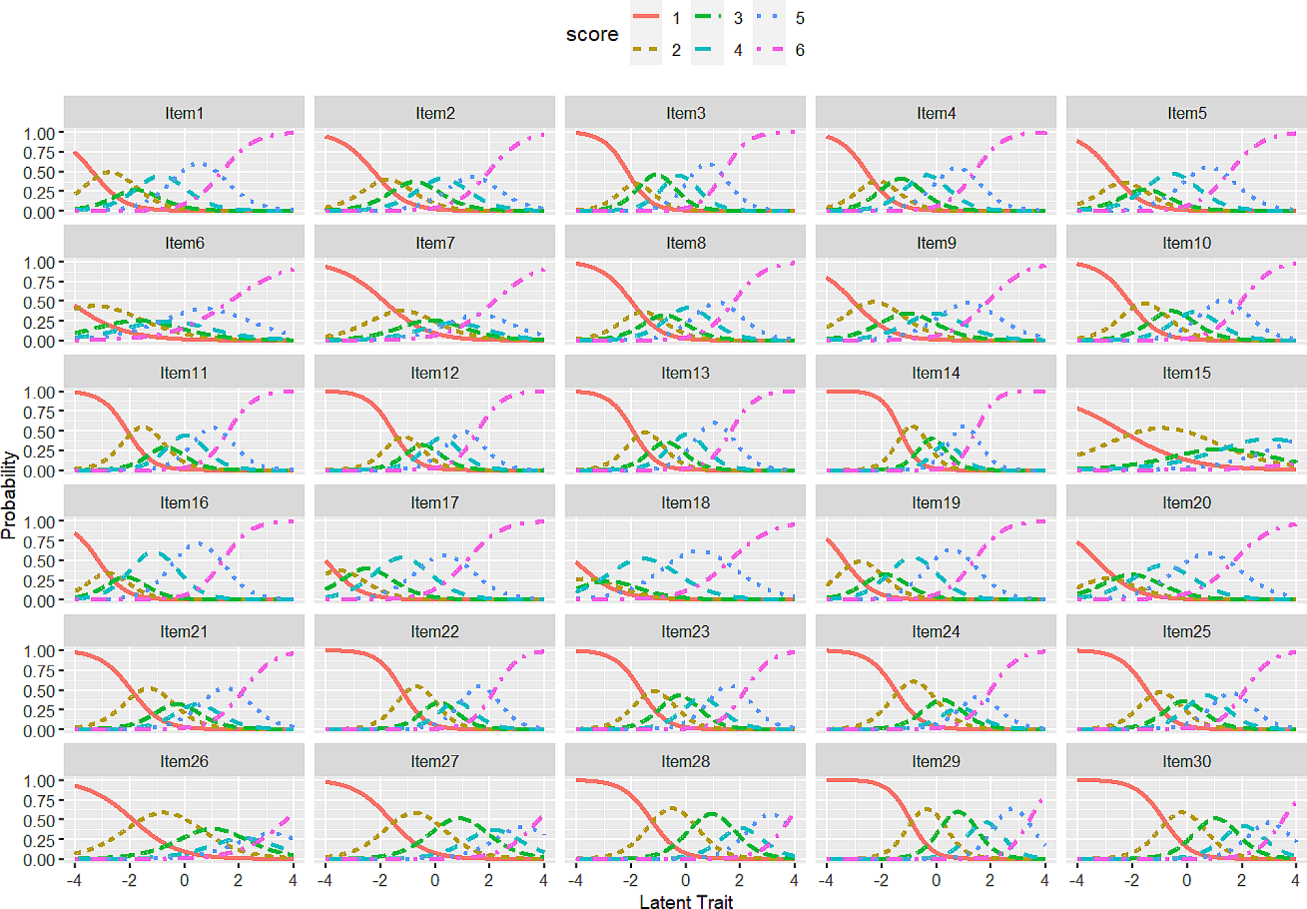
Item characteristics curves for the 30 items

The item characteristics curves are depicted in Fig. 1. This illustration highlights that items with a higher value of discrimination and a steeper curve slope tend to provide information about respondents’ EA within a narrow range [30]. For instance, consider item 14 (“I won’t do something until I absolutely have to”), which exhibits the highest discrimination (a = 2.95). In contrast, item 15 (“I try to deal with problems right away”) has the lowest curve slope and the lowest discrimination (a = 0.80).

### Item information curves (IIC)

**Fig. 2:**
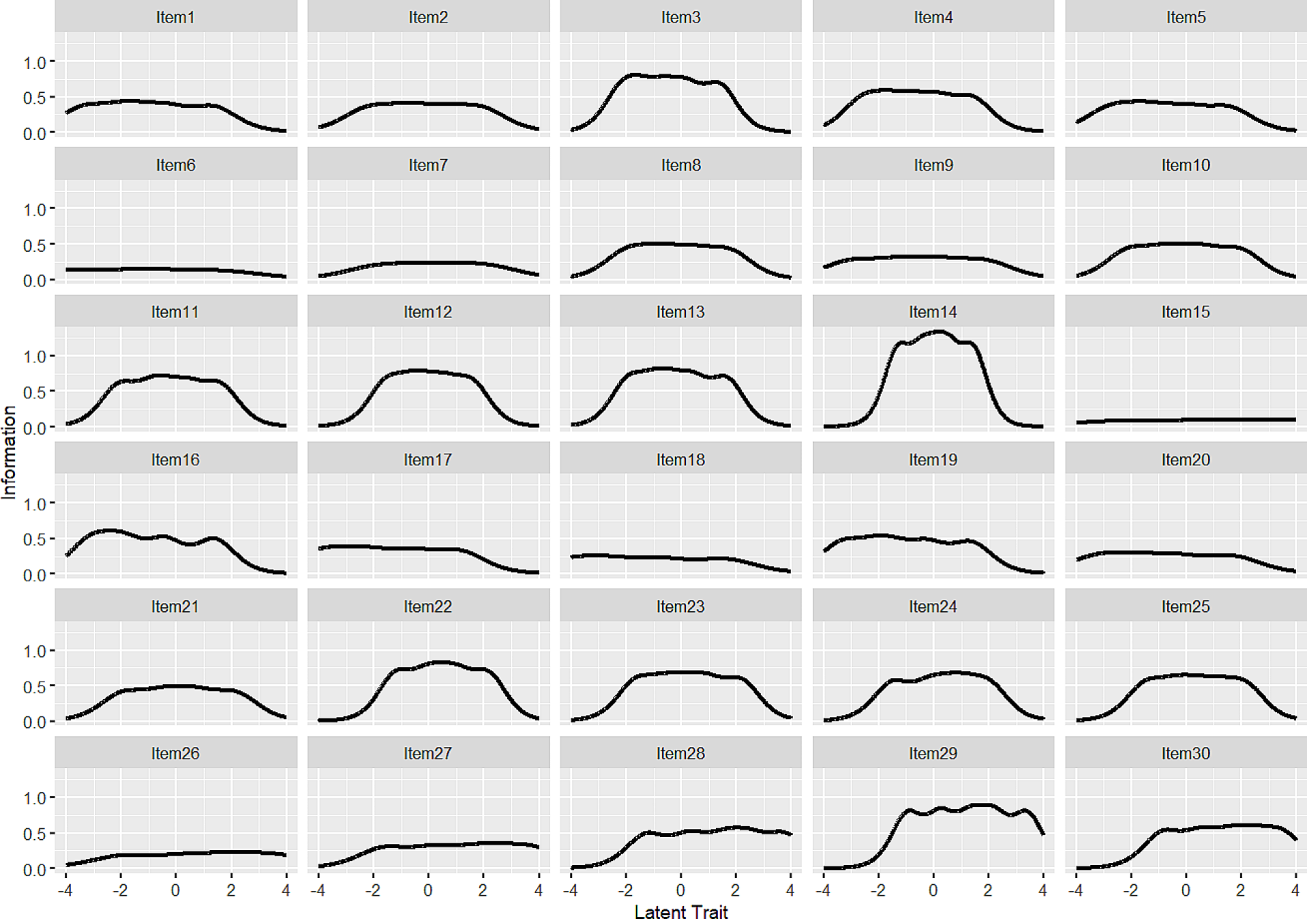
Item information curves for the 30 items

The IIC illustrates the amount of information that each item explains concerning the latent trait level [46]. As depicted in Fig. 2, item 14 (“I won’t do something until I absolutely have to”) provided the most information at the medium levels of the latent trait (θ = 0). In contrast, item 19 (“When unpleasant memories come to me, I try to put them out of my mind”) offered the most information at lower levels of the latent trait (θ = -2), while item 29 (“I don’t let gloomy thoughts stop me from doing what I want”) provided the most information at higher levels of the latent trait (θ = 2).

### Test information curves (TIC)

**Fig. 3:**
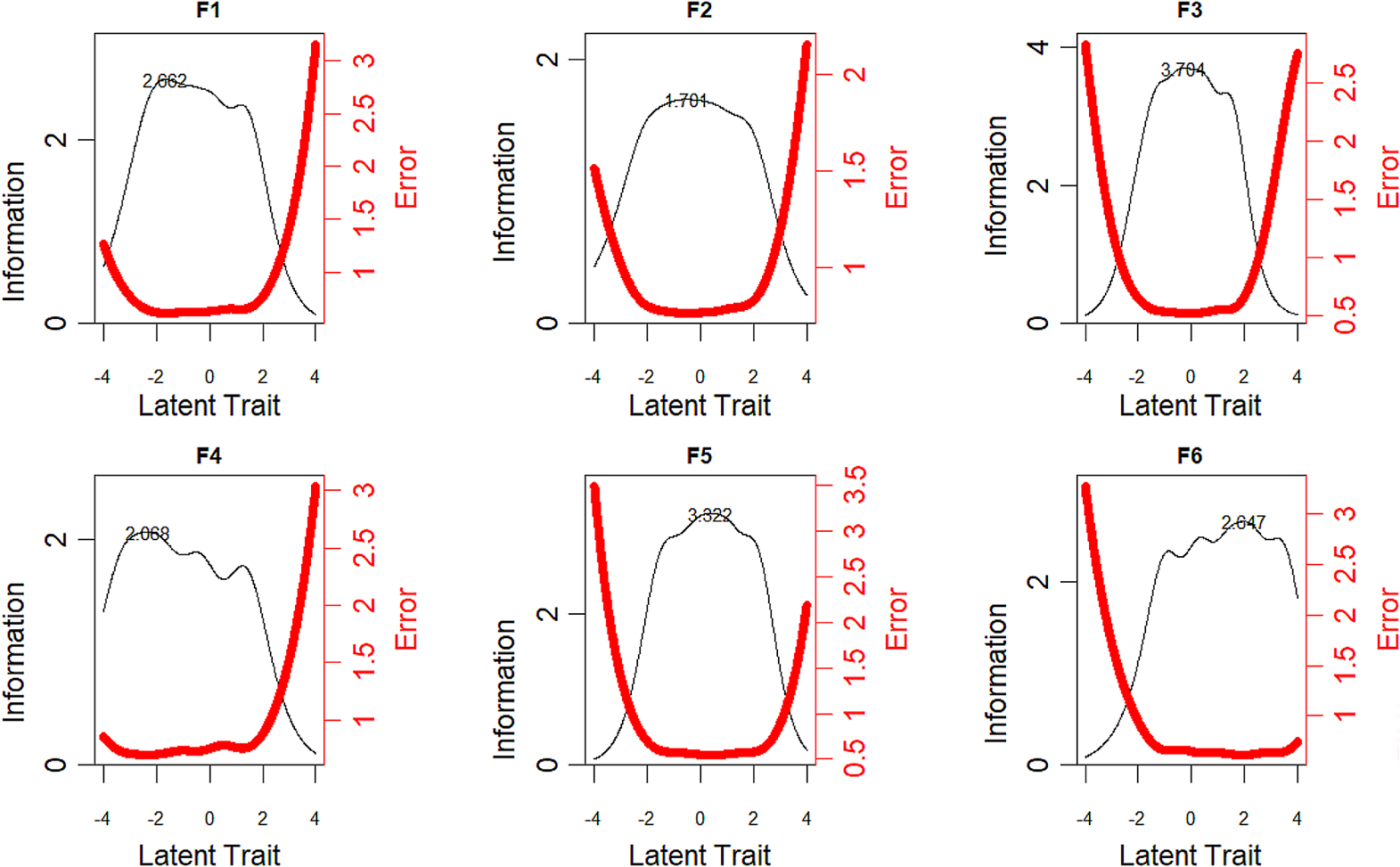
Test information curves of six subscales of MEAQ-30

The test information curves (TIC) were employed to evaluate the discriminative power of six subscales of MEAQ-30 (see Fig. 3). TIC indicated that different subscales provide the most information at varying levels of the latent trait. Specifically, behavioral avoidance (F1) and distraction/suppression (F4) provided the most information at lower latent trait levels and were more appropriate for measuring people with lower levels of EA. Conversely, distress endurance (F6) provided the most information at higher latent trait levels and was more suitable for measuring people with higher levels of EA. Lastly, distress aversion (F2), procrastination (F3), and repression/denial (F5) are more suitable for measuring people with moderate levels of EA.

## Discussion

This research evaluated the psychometric properties of the Chinese version of MEAQ-30 using both CTT and MIRT analysis. The two methods provided firm evidence for the reliability and validity of the Chinese version of MEAQ-30, indicating that it is a promising instrument for measuring EA among the Chinese population.

The present study demonstrated that the Chinese version of MEAQ-30 exhibits satisfactory structural validity and the same six-factor structure as the other language versions of the MEAQ-30 [20, 26]. Most items had standardized loadings above 0.5, which reflects the EA of the corresponding dimension well. Multiple confirmatory factor analyses supported the measurement invariance of MEAQ-30 across genders. This implied that the items of MEAQ-30 bear consistent meanings for both men and women, allowing for meaningful score comparisons. Furthermore, the internal consistency coefficients for the six dimensions of the Chinese version of MEAQ-30 exceed 0.7, indicating excellent reliability.

The current study showed that the Chinese version of MEAQ-30 had good convergent validity with the AAQ-II, neuroticism, depression, anxiety, and stress. The total MEAQ-30 score and the five dimensions were positively correlated with the above variables. Interestingly, this study found no significant correlation between scores on the distraction/suppression subscale and scores on anxiety and depression. This discrepancy may be interpreted from a cultural perspective. In Chinese culture, the suppression of emotions may be regarded as an adaptive behavior that reduces conflict and promotes social harmony [27, 47, 48]. Consequently, for some Chinese individuals, the distraction/suppression subscale items might be perceived as “not that negative” or “neutral”. They were more likely to agree with the formulations of these items (e.g., “When unpleasant memories come to me, I try to put them out of my mind”), potentially weakening the connection between distraction/ suppression and depression and anxiety.

Additionally, the Chinese version of the MEAQ-30 had better discriminant and incremental validity than the AAQ-II. There was a high correlation between AAQ-II and neuroticism (*r* = 0.82), consistent with previous research findings [14, 16], indicating that the concepts measured by AAQ-II and neuroticism overlap. Incremental validity tests showed that after controlling for the effects of neuroticism and AAQ-II on psychological distress, the MEAQ-30 remained an independent predictor variable for psychological distress. It explained a range of additional variance (5–8%) for depression, anxiety, and stress. These findings supported the validity of the Chinese version of MEAQ-30 for measuring EA.

This study proceeded to examine the item parameters of the Chinese version of MEAQ-30 through MIRT. The discrimination parameter of the 30 items ranged from 0.80 to 2.95, indicating that these items had moderate to high discriminatory power [49] and effectively discriminated between participants with different levels of EA. Furthermore, the difficulty parameter for most items ranged from − 3 to 3, indicating that the items were moderately difficult. Item 15 had the lowest discrimination and the highest difficulty. It provided the least information for procrastination, which is consistent with the results of CTT (e.g., the lowest item-total correlation and standardized factor loading). These results indicated that this item may need to be adjusted.

Item information curves highlighted that item 3, item 10, item 14, item 16, item 22, and item 29 provided higher information in assessing EA dimension. These items can effectively discriminate against individuals with different levels of EA. In contrast, item 2, item 6, item 15, item 18, item 21, and item 26 exhibited lower information in assessing the EA dimension. These six items had relatively low value in evaluating EA, and their item content may need to be improved. These findings suggested that the Chinese version of MEAQ-30 could be further optimized. Specifically, based on the analysis results of MIRT, the items in the MEAQ-30 could be screened to retain the more informative items, delete the less informative items, and further develop a briefer scale for assessing EA.

Furthermore, the test information curves evidenced the reliability of the six subscales of the MEAQ-30 for measuring the corresponding EA dimensions. It should be noted that the reliability and error of the EA measures vary depending on the dimension. Specifically, behavioral avoidance and distraction/suppression were more appropriate for measuring individuals with lower levels of EA (θ close to -2). As a result, the reliability of estimates is decreased for those with EA levels outside this range. In contrast, distress endurance provided the most information at higher levels of EA (θ close to 2), making this subscale more appropriate for measuring people with higher levels of EA. Additionally, distress aversion, procrastination, and repression/denial provided the most information at the moderate trait level (θ close to 0). These three subscales were more suitable for measuring people with moderate levels of EA. To sum up, these findings contributed to a better understanding of the performance of MEAQ-30 items across different dimensions.

### Implications

The current study provided solid evidence for the reliability and validity of the Chinese version of MEAQ-30 through CTT and MIRT, making it a valuable tool for assessing EA and opening avenues for further EA-related research in China. It is of significant importance to note that the comprehensive assessment of EA demonstrated by the MEAQ-30 facilitates the identification of potential associations between various avoidance behaviors and other outcomes related to mental health or psychopathology [18, 20]. Furthermore, the current findings highlighted the potential of MIRT to provide additional psychometric insights for MEAQ-30. These findings contributed to a detailed understanding of the item performance of the Chinese version of MEAQ-30.

#### Limitations and future studies

The current study had several limitations. First, the use of self-reporting alone may result in an overestimation of the correlation between variables, which could introduce common method biases [50]. Future work should consider validating the MEAQ-30 using diverse sources of information, including experimental designs and clinical ratings. Second, the participant’s sample was predominantly composed of college students. Given the limited number of professional employees in this study, no measurement invariance analysis was conducted between students and employees. Future studies should enhance sample diversity and explore the applicability of MEAQ-30 in other populations. Third, this study lacked a longitudinal assessment. Future longitudinal studies could investigate the causal relationships between EA and various variables.

## Conclusion

The current study provided firm evidence for the reliability and validity of the Chinese version of MEAQ-30 through CTT and MIRT. Consequently, the Chinese version of MEAQ-30 is a valuable tool for assessing experiential avoidance among Chinese populations.

### Electronic supplementary material

Below is the link to the electronic supplementary material.

Additional file 1

Additional file 2

## Data Availability

The data and materials are available from the corresponding author upon reasonable request.
