# Supplementary material for: Psychometric properties of the chinese version of multidimensional experiential avoidance questionnaire-30
Source: BMC Psychol. 2024 May 24;12:290. doi: 10.1186/s40359-024-01790-x (PMC11127356; doi:10.1186/s40359-024-01790-x)
Supplement: Supplementary file 1 — Additional file 1 [file 40359_2024_1790_MOESM1_ESM.docx]

**Appendix A**

**The English version of Multidimensional Experiential Avoidance Questionnaire-30**

| **Behavioral Avoidance** |
| --- |
| I won't do something if I think it will make me uncomfortable. |
| I avoid activities if there is even a small possibility of getting hurt. |
| If I am starting to feel trapped, I leave the situation immediately. |
| If I am in a slightly uncomfortable situation, I try to leave right away. |
| I avoid situations if there is a chance that I'll feel nervous. |
| **Distress Aversion** |
| If I could magically remove all of my painful memories, I would. |
| Happiness means never feeling any pain or disappointment. |
| One of my big goals is to be free from painful emotions. |
| I'd do anything to feel less stressed. |
| I would give up a lot not to feel bad. |
| **Procrastination** |
| I tend to put off unpleasant things that need to get done. |
| When I have something important to do I find myself doing a lot of other things instead. |
| I try to put off unpleasant tasks for as long as possible. |
| I won't do something until I absolutely have to. |
| I try to deal with problems right away (reversed item). |
| **Distraction/ Suppression** |
| When negative thoughts come up, I try to fill my head with something else. |
| When upsetting memories come up, I try to focus on other things. |
| I work hard to keep out of upsetting feelings. |
| When unpleasant memories come to me, I try to put them out of my mind. |
| When a negative thought comes up, I immediately try to think of something else |
| **Repression/Denial** |
| Others have told me that I suppress my feelings. |
| It's hard for me to know what I'm feelings. |
| It takes me a while to realize when I'm feeling bad. |
| I feel disconnected from my emotions. |
| People have told me that I'm not aware of my problems. |
| **Distress Endurance (reversed items)** |
| Even when I feel uncomfortable, I don't give up working toward things I value. |
| When I am hurting, I still do what needs to be done. |
| I don't let pain and discomfort stop me from getting what I want. |
| I don't let gloomy thoughts stop me from doing what I want. |
| When working on something important, I won't quit even if things get difficult. |

**Appendix B**

**The Chinese version of Multidimensional Experiential Avoidance Questionnaire-30**

| 项目 | 完全不符合 | 比较不符合 | 有点不符合 | 有点符合 | 比较符合 | 完全符合 |
| --- | --- | --- | --- | --- | --- | --- |
| 1.如果某件事会让我觉得不舒服，我就不会去做。 | 1 | 2 | 3 | 4 | 5 | 6 |
| 2.即使参加某个活动受伤的可能性很小，我也会避免参与。 | 1 | 2 | 3 | 4 | 5 | 6 |
| 3.如果我觉得自己陷入困境，我会马上离开。 | 1 | 2 | 3 | 4 | 5 | 6 |
| 4.如果遇到让我有点不舒服的情境，我会设法马上离开。 | 1 | 2 | 3 | 4 | 5 | 6 |
| 5.我会回避那些可能让我感到紧张的情境。 | 1 | 2 | 3 | 4 | 5 | 6 |
| 6.如果我能神奇地抹去所有痛苦的记忆，我会做的。 | 1 | 2 | 3 | 4 | 5 | 6 |
| 7.快乐意味着永远不会感到任何痛苦和失望。 | 1 | 2 | 3 | 4 | 5 | 6 |
| 8.我的一大目标是摆脱痛苦的情绪。 | 1 | 2 | 3 | 4 | 5 | 6 |
| 9.我愿意做任何事来减轻痛苦。 | 1 | 2 | 3 | 4 | 5 | 6 |
| 10.为了不感到痛苦，我愿意放弃很多。 | 1 | 2 | 3 | 4 | 5 | 6 |
| 11.我倾向于拖延那些令人不愉快但又需要做的事。 | 1 | 2 | 3 | 4 | 5 | 6 |
| 12.当我有重要的事情要做时，我发现自己却在做很多无关的事。 | 1 | 2 | 3 | 4 | 5 | 6 |
| 13.我尽可能长时间的拖延那些令人不快的事情。 | 1 | 2 | 3 | 4 | 5 | 6 |
| 14.一件事到了万不得已必须做时，我才会去做。 | 1 | 2 | 3 | 4 | 5 | 6 |
| 15.我会尽快处理遇到的问题^*^。 | 1 | 2 | 3 | 4 | 5 | 6 |
| 16.当消极的想法出现时，我试着用别的东西来占据大脑。 | 1 | 2 | 3 | 4 | 5 | 6 |
| 17.当令人沮丧的记忆出现时，我试着把注意力转到其他事物上。 | 1 | 2 | 3 | 4 | 5 | 6 |
| 18.我努力不让自己产生心烦意乱的感觉。 | 1 | 2 | 3 | 4 | 5 | 6 |
| 19.当不愉快的回忆出现时，我尽量把它们抛之脑后。 | 1 | 2 | 3 | 4 | 5 | 6 |
| 20.当一个消极的想法出现时，我会马上试着去想别的事。 | 1 | 2 | 33 | 4 | 5 | 6 |
| 21.其他人曾告诉我，我在压抑自己的情感。 | 1 | 2 | 3 | 4 | 5 | 6 |
| 22.我很难知道自己的感受是什么。 | 1 | 2 | 3 | 4 | 5 | 6 |
| 23.当我感觉糟糕的时候，我需要一段时间才能意识到。 | 1 | 2 | 3 | 4 | 5 | 6 |
| 24.我感觉和自己的情感脱节了。 | 1 | 2 | 3 | 4 | 5 | 6 |
| 25.人们曾告诉我，我没有意识到自己的问题。 | 1 | 2 | 3 | 4 | 5 | 6 |
| 26.即使我感到不适，我也不会放弃做我所重视的事情^*^。 | 1 | 2 | 3 | 4 | 5 | 6 |
| 27.当我感到痛苦时，我还是会做好该做的事^*^。 | 1 | 2 | 3 | 4 | 5 | 6 |
| 28.我不会让痛苦和不适阻止我得到我想要的^*^。 | 1 | 2 | 3 | 4 | 5 | 6 |
| 29.我不会让悲观的想法阻碍我去做我想做的事^*^。 | 1 | 2 | 3 | 4 | 5 | 6 |
| 30.在做重要的事情时，即使遇到困难，我也不会放弃^*^。 | 1 | 2 | 3 | 4 | 5 | 6 |

注：1-5，行为回避；6-10，痛苦厌恶；11-15，拖延；16-20，分心/抑制；21-25，压抑/否认；26-30，痛苦容忍。其中第15，26-30题为反向计分题目。
