# Supplementary figures and images for: Psychometric properties of the chinese version of multidimensional experiential avoidance questionnaire-30
Source: BMC Psychol. 2024 May 24;12:290. doi: 10.1186/s40359-024-01790-x (PMC11127356; doi:10.1186/s40359-024-01790-x)

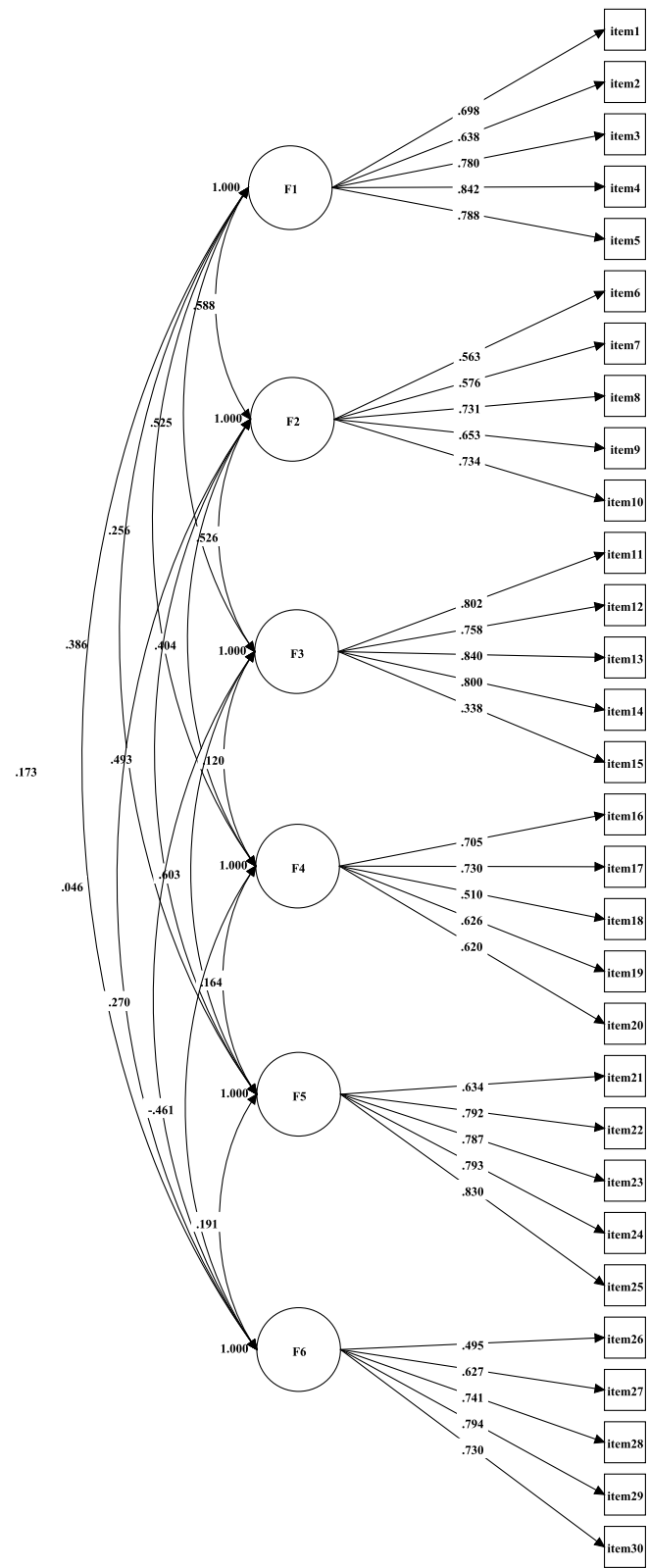

Supplement: Supplementary file 2 — Additional file 2 [file 40359_2024_1790_MOESM2_ESM.pdf]
